# Guided Application of Ventricular Catheters (GAVCA) - multicentre study to compare the ventricular catheter position after use of a catheter guide versus freehand application: study protocol for a randomised trail

**DOI:** 10.1186/1745-6215-14-428

**Published:** 2013-12-12

**Authors:** Andreas Schaumann, Ulrich-Wilhelm Thomale

**Affiliations:** 1 Department of Neurosurgery/Paediatric Neurosurgery, Campus Virchow Klinikum, Charité – Universitätsmedizin Berlin, Augustenburger Platz 1, 13353 Berlin, Germany

## Abstract

**Background:**

The standard technique for the placement of ventricular catheters (VC) comprises a high proportion of malpositioning of the catheter (12.5 to 40%). Technical advances such as neuronavigation or ultrasound have been shown to increase the accuracy of the procedure. Since these means result in significant technical and time consuming efforts, they are used for selected cases only. In order to simplify the controlled placement of ventricular catheters a newly developed smartphone assisted guiding tool has been introduced. In this study the efficacy and safety of this guiding tool is determined.

**Methods/design:**

This study is a multicentre, randomised, controlled trial. A total of 144 patients planned for an elective shunting procedure will be enrolled throughout 10 study centres within two years. The primary objective of the trial is to show the superiority of the guided placement in comparison to the standard freehand technique of ventricular catheter application. Patients will be followed up for 30 days after the operation in regard to image-based evaluation of the catheter position as well as possible shunt dysfunction and complications.

**Discussion:**

The Guided Application of Ventricular Catheters (GAVCA) trial compares the guided catheter positioning with the standard freehand technique of catheter placement in hydrocephalic patients. If superiority is shown, the standard technique may be changed with the advantage of a more reliable and safer positioning of the ventricular catheter with just a slight effort in time and pre-operative planning.

**Trial registration:**

The GAVCA trial is registered at ClinicalTrials.gov under the number NCT01811589.

## Background

The transcortical puncture of the ventricles is considered to be a simple neurosurgical procedure and is performed as one of the first procedures in neurosurgical training [1]. The ventricular catheter is considered as a lifelong functioning implant in the case of a shunting procedure; therefore special focus should be dedicated to the accurate positioning of the catheter as a correlation between catheter position and risk for proximal shunt obstruction is already described [2-7]. Several studies estimate the proportion of inaccuracy in the standard freehand technique to be in the range of 12.5 to 40% [2,4,8,9]. This freehand technique is based on the guidance by anatomical landmarks and the experience of the surgeon. In selected, more complicated cases technical advances such as neuronavigation or ultrasound might be used. However, this comprises high technical and time consuming efforts within a rather short procedure [4,10-13]. Seeing this problem, a more simplified technique is needed to be applied in a wide range of procedures. A mechanical catheter-guiding instrument was developed which enables a controlled placement of the catheters by applying one individual measure to the patients’ anatomy [14]. The instrument is suitable for a frontal pre-coronal approach. In earlier measurements based on MR images by using the foramen of Monro as a target, the angle towards the tangent of the skull surface was found to be relevant for the correct trajectory of puncture. Hereby, it could be shown that this angle is always rectangular in the sagittal plane whereas in the coronal plane an individual angulation must be measured respectively, with a mean deviation from rectangular of 1.96° ± 2.75° towards the medial margin up to 9.56° ± 4.14° towards the lateral margin of the ventricle [14]. That led to the development of the ventricular catheter guide which addresses a strict trajectory of a 90° angle to the sagittal surface of the skull and may be adjusted to an individual angle to the coronal surface. The measurements for the correct adjustment of the catheter guide are done on a coronal section of the cranium at a frontal paramedian entry point. Since regular DICOM viewing software does not allow an easy measurement of this parameter, this is facilitated in an iPhone application software (Version 1.1.1 for iOS6.1, Apple Inc., Cupertino, USA). Herein, the guiding instrument can virtually be visualised and placed on the skull of a coronal reconstructed section of the cranium for easy measurement of the trajectory’s angle towards the coronal surface of the skull.

The technical feasibility of the smartphone assisted guiding tool was verified in a single centre retrospective study [14]. In comparison to earlier studies on freehand ventricular placement, an advantage in the first pass cannulation and positioning was achieved.

The aim of the Guided Application of Ventricular Catheters (GAVCA) study is to show that the ventricular catheter guiding tool is superior to the standard freehand technique in terms of correct catheter position within the ipsilateral ventricle in a randomised multicentre trial.

## Methods/design

### Objectives

**Figure 1:**
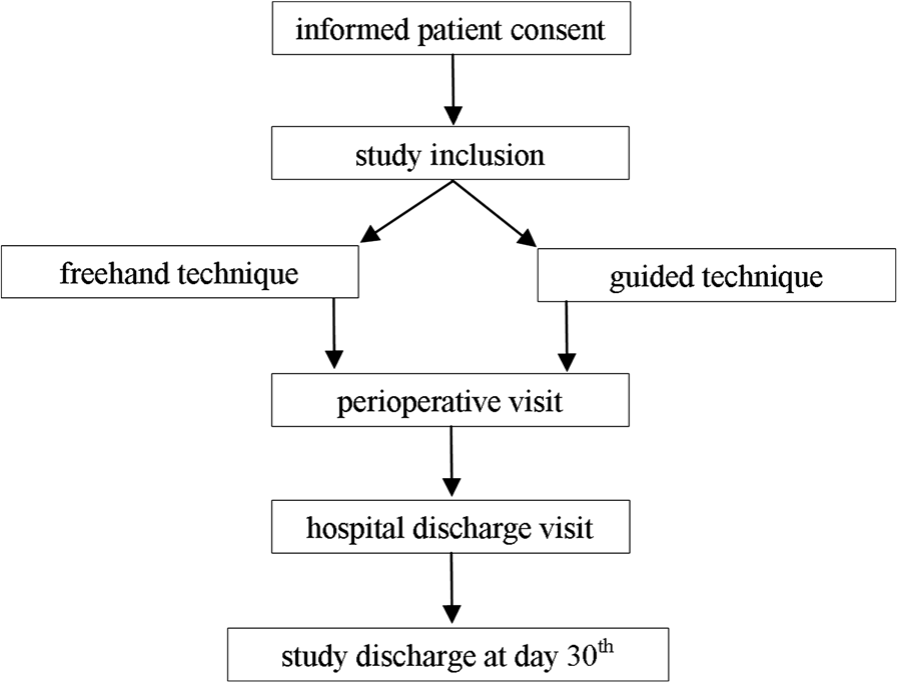
Study protocol flow chart representing the randomisation process.

This study is designed as a prospective, randomized, two-armed, multicentre trial comparing two surgical procedures (Figure 1). The primary objective of the study is to prove the superiority of the guided ventricular catheter placement (treatment arm 1) versus standard freehand technique (treatment arm 2) in terms of correct catheter position in the ipsilateral ventricle.

#### Primary endpoints

– the proportion of primary grade I or grade Ib catheter tip position in the ipsilateral ventricle

#### Secondary endpoints

– number of cannulation attempts until cerebrospinal fluid flow sets in

– the proportion of correct intraventricular localisation of the perforated catheter part

– early shunt failures

– complication rates (adverse events)

### Interventions

The procedure of cerebrospinal fluid shunting in hydrocephalus patients is an accepted neurosurgical standard. The placement of a ventricular catheter is a surgical skill that is taught early within neurosurgical training. Therefore, every neurosurgeon and neurosurgical resident is applicable to undertake the procedure within the trial as long as the handling of the catheter guide was taught within a standardised training.

The surgical technique is standardised as follows. The preparation of the skin flap is performed in order to expose at least 2 cm in diameter of the precoronal paramedian skull surface. One bore hole trepanation is performed at a localisation of 10 to 12.5 cm measured from nasion and 2 to 3.5 cm measured from midline in accordance to the individualised software measurements. The dura mater is to be coagulated and opened in order to enable the catheter puncture without being deviated at the dura level. The brain surface is punctiformly coagulated to open the leptomeningeal layers.

In treatment arm 1, the catheter guide is used with the individual parameters of coronal angulation to the skull surface and catheter length measured in a coronal section of the cranium with the catheter guide application software.

In treatment arm 2, the catheter placement is undertaken in freehand technique.

In both arms, the catheter is fixed by a rectangular redirector of the catheter from the bore hole to the subcutaneous tissue, such as a bore hole reservoir or a 90° connector.

In case of an unsuccessful cannulation, the surgeon will re-try the puncture in the freehand group. In the guided catheter group, the surgeon will repeat the use of the guide in case any mistakes in its application are detected and may need to be corrected, otherwise a crossover to the freehand technique will be performed.

### Follow-up

At the day of surgery relevant perioperative parameters are reported. This includes, for both treatment arms, the age, sex, the underlying cause for hydrocephalus, the ventricular width according to the frontal occipital horn ratio (FOHR) and the frontal occipital width ratio (FOHWR), the personal and the device-specific expertise level of the operating surgeon, the number of cannulation attempts, the type of ventricular catheter, the localisation of the entry point, the time of surgery, and any adverse events.

The individual angulation of the catheter guide and the length of the implanted catheter are reported for the treatment arm as measured in the ventricular guide application software. After surgery a MRI-scan or CT-scan as postoperative control is performed to document the catheter position and any adverse events, such as intra-cerebral haemorrhage. The follow-up examinations will be performed on the day of discharge from hospital and 30 to 40 days after surgery in order to report catheter dysfunction with the need of a re-operation and serious adverse events (Table 1).

**Table 1:** Study protocol representing the relevant CRF visits

**Method**	**Visit 0**	**Visit 1 pre-OP**	**Visit 2 peri-OP**	**Visit 3 discharge**	**Visit 4 end of study**	**CT catheter-control**
**screening, consent, inclusion**	x					
**randomisation, patient data**		x				
**surgery**			x			
**perioperative study visit, complications**			x			
**study visit, complications, early shunt failure**				x	x	
**postoperative brain scan (until study visit 4)**						x

### Rating of the catheter position

**Figure 2:**
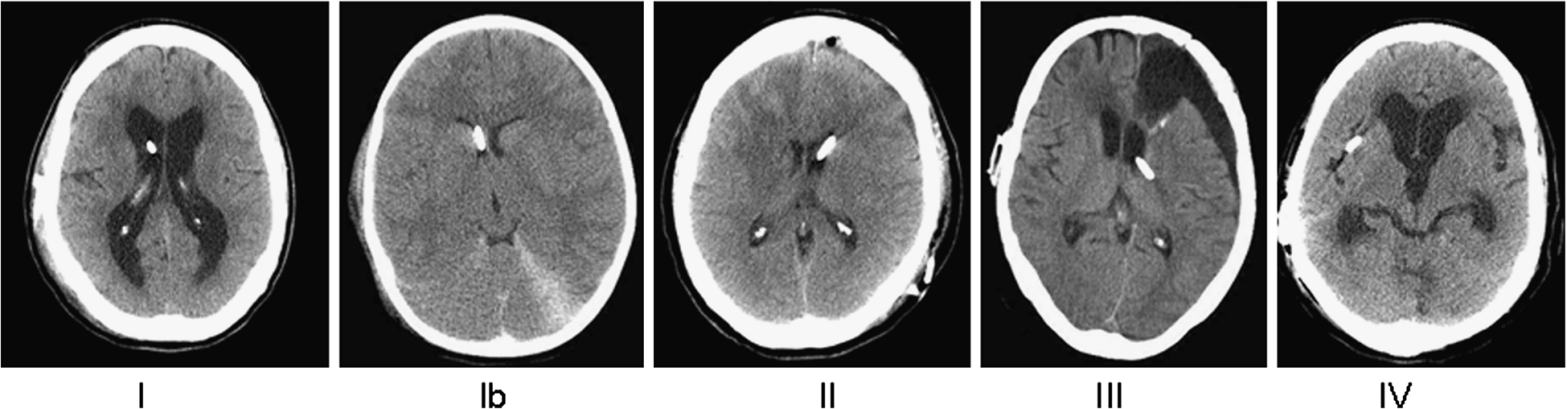
**Catheter position rating scale. Grade I**: ideal catheter position with contact to the ventricular wall less than 0.5 cm. **Grade Ib**: contact to the medial and lateral ventricular wall in the case of ventricular width equals the catheter diameter. **Grade II**: contact to the ventricular wall or the choroid plexus of more than 0.5 cm. **Grade III**: only partial intraventricular position of the catheter (less than 1.5 cm intraventricular). **Grade IV**: extraventricular position of the catheter (less than 0.5 cm intraventricular).

The catheter position is rated in a centralised manner in regard to the 2 cm tip of the catheter (perforated part) by a neuroradiologist. The neuroradiologist is blinded for the treatment arm and outcome of the patient and evaluates the position of the catheter on the postoperative reconstructed MRI or CT scans, which were performed within 30 days after surgery (Figure 2). The rating will be performed using the following protocol [15].

– **grade I**: ideal catheter position without contact to the ventricular wall of more than 0.5 cm

– **grade Ib**: contact to the medial and lateral ventricular wall in the case the ventricular width equals the catheter diameter

– **grade II**: contact to the ventricular wall or the choroid plexus of more than 0.5 cm

– **grade III**: partly intraventricular position of the catheter (less than 1.5 cm intraventricular)

– **grade IV**: extraventricular position of the catheter (less than 0.5 cm intraventricular)

In addition, the postoperative image will be evaluated for the position of the respective catheter tip in the ipsilateral, contralateral, third ventricle or in any extraventricular compartment. Therefore the length of the intraventricular catheter part will be measured. Compared to preoperative ventricular width values, the postoperative FOHR and FOHWR will also be measured. As adverse events in imaging, any possible bleeding or air inclusions will be detected.

### Study tool

**Figure 3:**
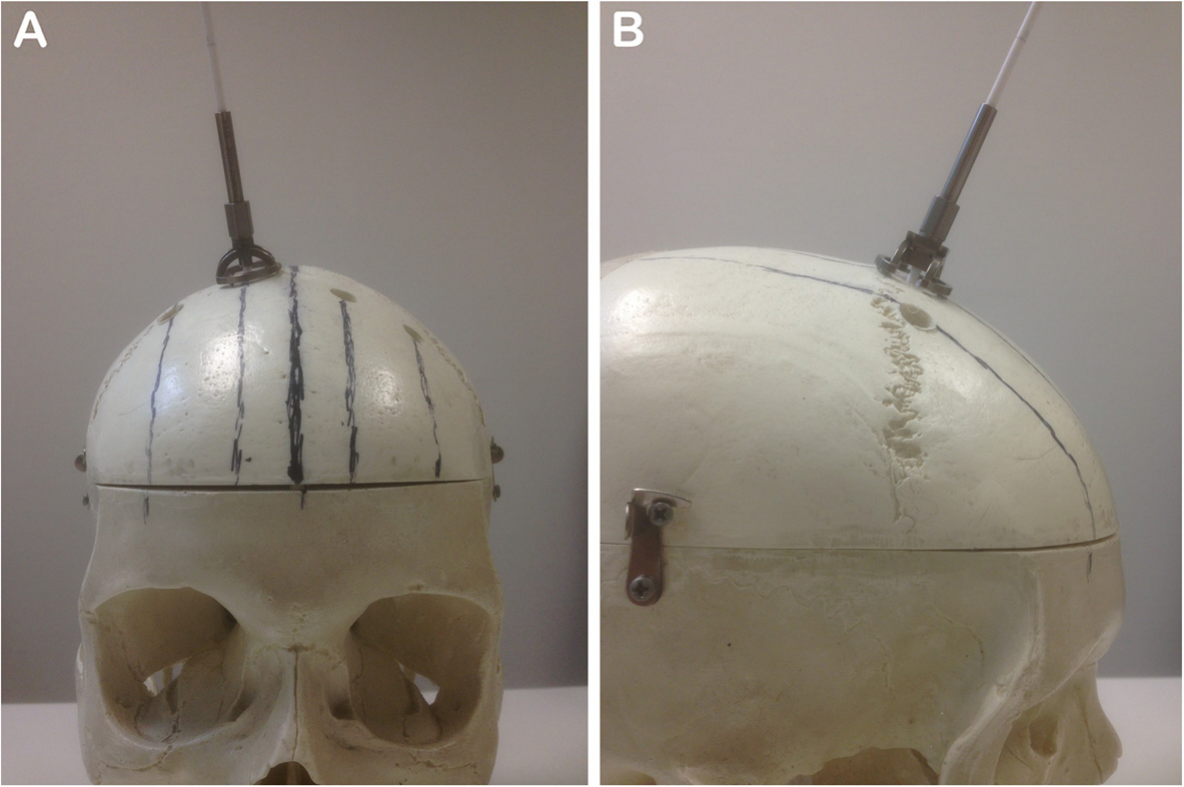
**Ventricular catheter guide placed on the surface of the skull model as used during surgery. A**: In the coronal plane, the individual angulation towards the skull surface can be adjusted. **B**: In the sagittal plane, the angulation to the skull surface is fixed at 90°.

The catheter guide is a medical instrument, which is approved and bears the CE-Mark (Thomale Guide, certificate registration number: 009066 MR2) It is produced by the Miethke company (Potsdam, Germany) and distributed by Aesculap, Inc. (Tuttlingen, Germany). The catheter guide allows an individual angulation in the coronal plane whereas the angulation in the sagittal plane is in a strict rectangular direction (Figure 3).

### Study software

**Figure 4:**
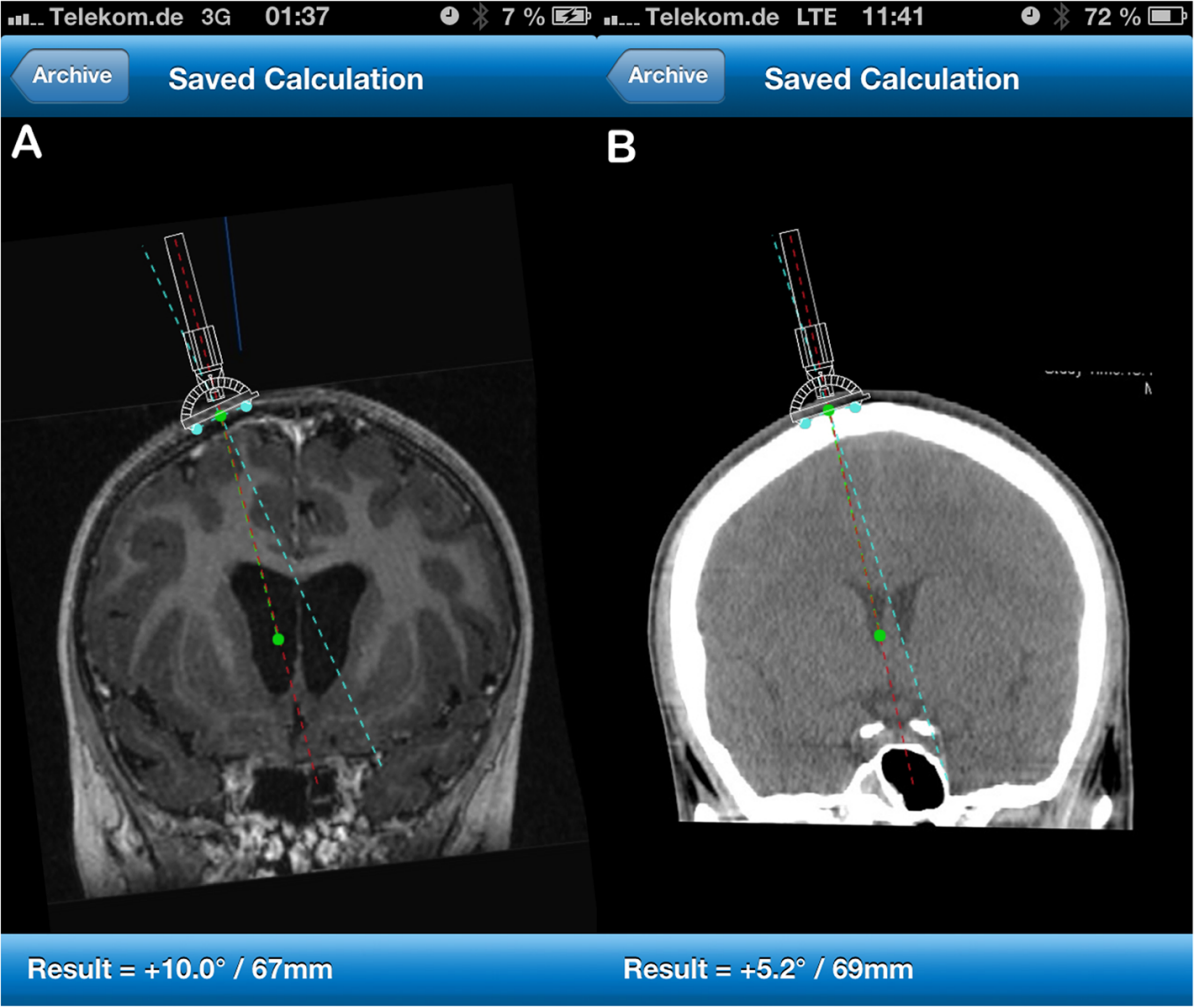
**Measurements acquired through the smartphone application. A**: Coronal MRI section with moderate enlarged ventricles with virtual placement of the guide on the skull surface. Angulation of catheter trajectory (red dotted line) is measured at 10° deviation from 90° (light blue dotted line) tilting the guiding tube towards the midline. The catheter length (green dotted line) is measured at 67 mm. **B**: Coronal CT reconstruction along body axis with the measurements revealing an angulation of 5.2° deviation from rectangular with the guiding tube tilt towards the midline and a catheter length of 69 mm.

In order to identify the ideal trajectory in the coronal plane an image processing software (https://itunes.apple.com/en/app/thomale-guide-application/id648839804?mt=8 Thomale Guide App, Version 1.1.1 for iOS 6.1, Apple Inc., Cupertino, USA) was developed for the iPhone/iPad, which is also approved in the EU (certificate registration number: 009066 MR2). This software can be downloaded for free from the Apple app store [14]. The application is used to measure the angle of the trajectory, the catheter length and the position of the bore hole relative to midline from imported images (Figure 4). Alternatively, the individual patient’s parameters can also be obtained by processing on an adequate DICOM-viewer-software (Centricity, Gentricity 3.0, General Electrics Inc., Fairfield, USA), if the user is familiar with that.

### Trial population

The trial population will be recruited consecutively from the hydrocephalic patients who are scheduled for a shunting procedure in the participating medical centres. The participation in the trial ends with the final visit at Day 30 after surgery. Further follow-up visits are not scheduled. A detailed overview of the eligibility criteria are given as follows.

#### Inclusion criteria

– hydrocephalic patient needing a shunting procedure

– fronto-occipital horn ratio (FOHR) <0.5 [16]

– positioning via a mature brain tissue trajectory

– pre-coronal/frontal approach to the ventricle

– signed informed consent

#### Exclusion criteria

– previous known uneven bone surface at the site of the approach

– slit ventricles with an fronto-occipital horn width ratio (FOHWR) <0.05 [17]

– participation in other clinical trials with interfering endpoints

– patients unable to give informed consent

### Sample size

The sample size was determined to verify the hypothesis of superiority of treatment arm 1 (guided catheter placement) in comparison to treatment arm 2 (freehand technique) in regard to the proportion of primary cannulation of grade I or grade Ib in the ipsilateral ventricle. A successful catheter positioning of grade I or Ib in the ipsilateral ventricle is regarded as a positive response.

As we calculate with a 5% non-responder rate in treatment arm 1, the positive response in treatment arm 1 was reduced to 78.8% according to the weighted average. The difference between the two balanced patient cohorts is rated on a one-sided Chi-squared test with a significance of 2.5% and a power of 80%. Sixty-five patients are to be enrolled in each treatment arm as calculated with the software STATA 12.1, (StataCorp, College Station, TX, USA). Including a 10% dropout rate, the number increases to 72 patients per treatment arm.

### Randomisation and blinding

Patients’ allocation to both study arms is balanced (1:1). In order to minimise the size differences between groups, blocks of random length are used. The randomisation system is integrated into a web-based, password protected electronic case report form (http://www.studiesportal.com, Aesculap, Inc., Tuttlingen, Germany). Blinding of the operating surgeon is not feasible. The response (catheter position) is rated on the postoperative scans by the treatment-blinded outcome evaluator, which is an experienced neuroradiologist.

### Statistical analysis

#### Primary variable

The proportion of patients with a primary placement of grade I or Ib position of the catheter in the ipsilateral ventricle, as well as associated 95% confidence intervals, will be reported for each treatment arm. The study null hypothesis, which is to reject, states that the catheter guided treatment arm is equal or inferior to the freehand technique arm regarding the proportion of correct catheter positioning in the ipsilateral ventricle. This null hypothesis has to be disproved to state the superiority of the catheter guide treatment.

*H 0*: *P*_*TG*_ – *P*_FH_ < = 0 (*P*_*TG*_ < =*P*_*FH*_);

*H 1*: *P*_TG_ – *P*_*FH*_ > 0 (*P*_*TG*_ > *P*_*FH*_).

*P* is equal to the responder rate (TG – Thomale Guide, FH – freehand)

The analysis will be based on the intention-to-treat population in order to include catheter guide treatment problems with a change in the intraoperative treatment of the patient (cross-over). In the case of non-applicability of the catheter guide (for example, uneven bony surface) in patients randomised into the catheter guide treatment arm, they will be treated according to the freehand technique treatment arm. This is also applicable for patients with unsuccessful cannulation of the ventricle in the catheter guide treatment arm according to the decision of the operating surgeon. In the intention-to-treat analysis, all these cross-over patients will be analysed in the catheter guide treatment arm.

In a secondary step, a per-protocol reporting will be done, which excludes cross-over patients.

In a tertiary analysis as-treated reporting (safety evaluation) is foreseen.

#### Secondary variables

The secondary variables (number of cannulation attempts, the proportion of correct intraventricular positioning of the perforated part of the catheter and the complication rate/adverse events) will be reported descriptively based on an intention-to-treat as well as the per-protocol population. The proportion of “early-shunt-failures” as being a safety-parameter will be reported according to the as-treated population.

#### Level of significance

The one-sided test level for the confirmatory test is 2.5%. The explorative tests will be performed on a two-sided level of 5%. To adjust for multiple effects and covariates without running multiple tests, appropriate multivariate regression models will be used for analysis.

### Trial organisation

The trial is initiated and designed by the investigators. It is funded by B. Braun Aesculap. The study is conducted in 10 German medical centres (see the list at the end of this paper). These study centres were chosen for their experience in treatment of hydrocephalic patients and their willingness to adhere to the study protocol.

### Data management and monitoring

The clinical data will be reported on an electronic case report form (online) by the investigators at the study site. The access to the web-based database is individualised and password protected. The study shall be conducted according to the ISO 14155 guideline for medical device studies. Study monitoring is performed by an authorised and qualified representative of the study administration.

### Ethical aspects

The trial is approved by the ethics committee of the Charité - Universitätsmedizin Berlin. Secondary approvals are obtained from the ethics committee of each study centre. The trial is conducted in agreement with the principles of the Declaration of Helsinki and the German law for medical devices. Patients are enrolled into the trial only after a signed informed consent is obtained.

## Discussion

The placement of ventricular catheters is a relatively easy and straight-forward procedure, for which the neurosurgeons are trained during their first years of residency [1]. It may be the most often performed procedure in neurosurgery, since it becomes necessary in patients with cerebrospinal fluid drainage blockage, as transient external drainage before larger tumor surgeries, for intracranial pressure monitoring, for example, in severe traumatic brain injury patients, and in hydrocephalus patients for cerebrospinal fluid (CSF) shunting [3,5]. In all of these indications the accuracy of the ventricular catheter placement will correlate with the proper functioning of the catheter [2,6]. In the shunted hydrocephalus patients the catheter is part of a lifelong implant, thus, accurate care must be delivered for proper placement and long-term functioning of the implant [2]. This is regularly performed in freehand technique according to standardised anatomical landmarks. Recent studies have shown that this technique results in a 12.5% to 40% inaccuracy of catheter placement [2,4,8,9]. Thus, in selected cases, technical efforts, such as neuronavigation and ultrasound, were used to enhance the proportion of accuracy [3,7,11,12,16]. The reason for not using it in all cases is that the preparation time of technical advances is too long in relation to the procedure itself and the availability in each centre for these procedures are limited [12]. Another aspect is a limited awareness of neurosurgeons concerning the failure rate of accurate positioning of the catheter in a rather simple procedure. This holds especially true since the failure of a catheter which is inaccurately placed will happen within an interval when the correlation to the initial procedure cannot be drawn anymore. A survey among neurosurgeons about the use of technical efforts for the accurate placement of ventricular catheter disclosed that 30% did not want to enhance the freehand method while roughly 60% would use technical advances if they would not prolong the surgery by 5 or 10 minutes [10]. With these conditions, we recently introduced a mechanical instrument for guiding ventricular catheter placement. For this technique, one individual parameter is necessary to be measured in preoperative imaging and applied to this instrument to achieve controlled and accurate puncture of the ventricle via a frontal pre-coronal approach. The safety and efficacy of this technique was shown in a single centre study so far [14]. The proposed protocol is designed to prospectively test the method on the larger basis of a multicentre randomised trial.

Our own retrospective analysis of inaccurate placement of the ventricular catheter reveals that the inaccuracy correlates with smaller ventricles. We found that an FOHR of smaller than 0.5 resulted in 15% catheter malpositioning at our institution. This cutoff measure represents a ventricular size in which regularly no technical help is applied for better accuracy. The catheter guide is thus meant to be used in a routine manner even if the ventricular size seems not necessarily to be challenging, but to achieve a higher level of reliability of the lifelong implanted ventricular catheter.

The technique itself incorporates innovatively the good availability of smartphones as a measurement tool for the surgical instrument. Hereby, an application software was designed in order to easily apply the measurements in one coronal brain section image. Virtual visualisation of the instrument does help to perform the measurements intuitively and to safely apply the measures to the instrument and the patient.

The image processing can also be done on every advanced DICOM viewer, which allows in-picture-angle-measurements. However, we experienced herewith a more complicated workflow and a higher risk that the measurements are not accurately transferred to the patient. The images used for the smartphone application must be coronal sections where the frontal horns are depicted [14]. Herein, the trajectory will be projected from oblique to strictly coronal. This can be transferred as an anonymous image file, for example, via e-mail to the smartphone and may then be integrated in the software application. When higher accuracy is needed, the image section can also be reconstructed along the virtual trajectory of the catheter placement using a navigation-software. This will enable the measurements to be done at the exact localisation of the entry point. In more difficult cases, such as slit ventricles (FOHWR <0.05), a neuronavigation system for ventricular catheter positioning might still be advantageous.

According to our initial data, the catheter guide used in combination with the iPhone application revealed a good reliability in the pilot study. Here, the catheter guide was used in narrow ventricles with a mean FOHR of 0.38+/−0.05. All catheters could be placed in a functional correct position with primary cannulation. In 8%, the contralateral ventricle was punctured, which was observed within the first 10 patients, so a learning curve in using the catheter guide must be stated [14].

The catheter guide has to be used directly on the bony surface to ensure the angulation be applied in relation to the surface of the skull. That may limit the use of the catheter guide when insufficient bone area is exposed or the surface may be uneven due to previous surgeries at the site of the entry point. Transcutaneous cannulation of the ventricles, for example, in the case of an external ventricle drainage as performed on intensive care units, are not applicable with the current design of the tool.

The placement of ventricular catheters is a frequent procedure in Neurosurgery which is regularly performed in freehand technique. As recent literature has reported this is not sufficient for lifelong implants as ventricular catheters are used in CSF shunts for hydrocephalus patients. An easy to use surgical instrument used together with a smartphone software application is tested within the current study design in a prospective randomised multicentre fashion in order to evaluate its safety and efficacy for the routine use in a wide range of patients.

### Participating hospitals

– Charité Universitätsmedizin Berlin, Department of Neurosurgery, Berlin

– Klinikum Kassel, Department of Neurosurgery, Kassel

– Unfallkrankenhaus Berlin, Department of Neurosurgery, Berlin

– Eberhard Karls University Tübingen, Department of Neurosurgery, Tübingen

– Ruprecht Karls University Heidelberg, Department of Neurosurgery, Heidelberg

– Georg August University Göttingen, Department of Neurosurgery, Göttingen

– Dietrich-Bonhoeffer-Klinikum, Department of Neurosurgery, Neubrandenburg

– Heinrich Heine University Düsseldorf, Department of Neurosurgery, Düsseldorf

– University Duisburg Essen, Department of Neurosurgery, Essen

### Current status

A first investigators’ meeting was held in May 2012, where the key points of the study design were discussed and agreed on by all clinical trial centres. In November 2012, the study protocol was then completed and the trial was approved by the ethics committee of the Charité - Universitätsmedizin Berlin on 18 February 2013. On 8 April 2013 the first patient was enrolled in the GAVCA trial. The estimated patient recruitment time for 144 patients in 10 study centres is 24 months. At the time of submission, 24 patients were recruited in 5 out of 10 participating centres.

## Abbreviations

CSF: Cerebrospinal fluid; GAVCA: Guided Application of Ventricular Catheters; FOHR: Frontal occipital horn ratio; FOHWR: Frontal occipital width ratio; VC: Ventricular catheters.

## Competing interests

AS has no competeing interests. UWT holds the patent for the Guide instrument. UWT receives compensation for speaker activities from Aesculap Inc.

## Authors’ contribution

AS and UWT designed the study conception and protocol as well as drafted the manuscript. Both authors have read and approved the manuscript.
